# 4378 Dynamic Influences on Population Health Management by Asthma Community Health Worker (CHW) Programs: An Agent Based Modeling Approach

**DOI:** 10.1017/cts.2020.385

**Published:** 2020-07-29

**Authors:** Bradley Kramer, Jessica Jones-Smith, Bryan Weiner

**Affiliations:** 1 University of Washington

## Abstract

OBJECTIVES/GOALS: Twenty years of evidence show CHW home-visits for asthma improve population health and lower overall health care system costs by reducing costly Emergency Department (ED) visits. We built a model to communicate these results to decision makers and demonstrate how program modifications can improve CHW program sustainability and long-term cost savings. METHODS/STUDY POPULATION: This CHW program simulation model (CHWsim) examines program level outcomes of sustainability and costs. CHWsim is populated with individuals in an asthma registry with frequent ED visits. The simulation shows the uptake of the CHW program, and the less frequent use of ED visits based on empirical data from a recent randomized controlled trial (RCT). CHWsim is interactive, allowing for parameter adjustments to programming and robust quantitative evaluation of those changes. We study sustainability using parameters based on number of CHWs, case load, frequency and duration of program. For cost outcomes, we use empirical data from a published return on investment study to demonstrate the cost of CHW programming vs. health care systems savings from reduced ED visits. RESULTS/ANTICIPATED RESULTS: We demonstrate a basic model that successfully simulates a recent RCT (n = 551), replicating the primary outcome of reduced ED visits within the Confidence Intervals. The model is validated by reproducing results of other RCTs. Results will also be presented from an in-process expanded model that simulates the intervention in a broader population (n = 4000). We test two programmatic changes and demonstrate how these modifications might improve health outcomes (reduced ED visits) that translate into cost savings to the system. 1) In the original trial, we served only one household member; in the model, we treat the full household, serving several individuals at the same time. 2) We also consider the assumption that a short annual visit might sustain the known 12-month health effect across many years. DISCUSSION/SIGNIFICANCE OF IMPACT: CHWsim uses individual level local data and patient characteristics to demonstrate the impact of program efficiencies to improve CHW program sustainability and reduce health care system costs without expensive new RCTs. CHWsim has the potential to improve CHW program delivery and influence funders to provide support.

